# The Need for Consultation

**Published:** 1923-11

**Authors:** 


### The Need for Consultation.

SIR,—As this dear old country of ours rumbles along, those to whom we entrust our lives and destinies get excited every now and then over some particular mischief, and proceed to create new local authorities, or to scatter fresh duties about, and almost certainly to appoint some new kind of inspector. The local authorities then get excited from time to time and appoint large and expensive deputations to go to London, or to take joy-rides to some delectable spot where Conferences are being held.

Heaven forbid that anyone should suggest more Conferences, but I would humbly enquire whether there is not need for more conference, without the capital C, especially in regard to health measures. Great assemblies are singularly ineffective. But we are learning a little in these days of the value of "regional" activity. And I suggest that neighbouring county authorities and borough authorities could do much useful work if they would from time to time confer as to the results of their schemes and organisations. Platitudes from the platform do not move things along much, but if the Chairman of the Health Committee and the Medical Officer of one area were to meet their "opposite numbers" for three or four other areas in a patient and earnest endeavour to compare notes as to results, and discuss frankly where failure lay, and where success, where economy was called for, and where development, some really valuable results might ensue.

I have more particularly in mind questions relating to the schemes instituted in the last ten or fifteen years for the treatment of tuberculosis, venereal disease, and maternity and child welfare. But over the whole field of health activities there is need for frank examination from time to time of such questions as: What plan are we working upon? In what direction are we moving? Are we indeed merely drifting?

If any of the health authorities who are reading your paper in increasing numbers find anything of value in my suggestion, and will forgive my remark about the joy-rides, I shall feel justified in having asked for space for this communication. If our heavy expenditure on health matters is wisely directed, and modified or extended as sage experience dictates, I for one do not mind paying my rates without grumbling. And I venture, therefore, to subscribe myself A CHEERFUL RATEPAYER.

